# *Plasmodium berghei* Circumvents Immune Responses Induced by Merozoite Surface Protein 1- and Apical Membrane Antigen 1-Based Vaccines

**DOI:** 10.1371/journal.pone.0013727

**Published:** 2010-10-28

**Authors:** Shigeto Yoshida, Hiroshi Nagumo, Takashi Yokomine, Hitomi Araki, Ayaka Suzuki, Hiroyuki Matsuoka

**Affiliations:** Division of Medical Zoology, Department of Infection and Immunity, Jichi Medical University, Tochigi, Japan; BMSI-A*STAR, Singapore

## Abstract

**Background:**

Two current leading malaria blood-stage vaccine candidate antigens for *Plasmodium falciparum*, the C-terminal region of merozoite surface protein 1 (MSP1_19_) and apical membrane antigen 1 (AMA1), have been prioritized because of outstanding protective efficacies achieved in a rodent malaria *Plasmodium yoelii* model. However, *P. falciparum* vaccines based on these antigens have had disappointing outcomes in clinical trials. Discrepancies in the vaccine efficacies observed between the *P. yoelii* model and human clinical trials still remain problematic.

**Methodology and Results:**

In this study, we assessed the protective efficacies of a series of MSP1_19_- and AMA1-based vaccines using the *P. berghei* rodent malarial parasite and its transgenic models. Immunization of mice with a baculoviral-based vaccine (BBV) expressing *P. falciparum* MSP1_19_ induced high titers of PfMSP1_19_-specific antibodies that strongly reacted with *P. falciparum* blood-stage parasites. However, no protection was achieved following lethal challenge with transgenic *P. berghei* expressing PfMSP1_19_ in place of native PbMSP1_19_. Similarly, neither *P. berghei* MSP1_19_- nor AMA1-BBV was effective against *P. berghei*. In contrast, immunization with *P. yoelii* MSP1_19_- and AMA1-BBVs provided 100% and 40% protection, respectively, against *P. yoelii* lethal challenge. Mice that naturally acquired sterile immunity against *P. berghei* became cross-resistant to *P. yoelii*, but not vice versa.

**Conclusion:**

This is the first study to address blood-stage vaccine efficacies using both *P. berghei* and *P. yoelii* models at the same time. *P. berghei* completely circumvents immune responses induced by MSP1_19_- and AMA1-based vaccines, suggesting that *P. berghei* possesses additional molecules and/or mechanisms that circumvent the host's immune responses to MSP1_19_ and AMA1, which are lacking in *P. yoelii*. Although it is not known whether *P. falciparum* shares these escape mechanisms with *P. berghei*, *P. berghei* and its transgenic models may have potential as useful tools for identifying and evaluating new blood-stage vaccine candidate antigens for *P. falciparum*.

## Introduction

Malaria is an enormous public health problem worldwide and kills one to two million people every year, mostly children residing in Africa. Clearly, an effective vaccine for the control of malaria is urgently needed. The selection of protein antigens for malaria vaccine development has been hampered by the lack of a reliable and readily accessible challenge system for *Plasmodium falciparum*. Accordingly, much attention has focused on the study of laboratory rodents infected by murine malaria parasite species, most notably *P. yoelii* and *P. berghei*. Although not perfect models for human infection, these systems have proved useful, and important advances in our understanding of the principles of vaccine design have followed their use. For blood-stage vaccine development, in particular, the *P. yoelii*-murine model has greatly contributed to the evaluation of protective efficacies of blood-stage antigens prior to human clinical trials. Based on the *P. yoelii* model, many asexual blood-stage candidate antigens have been identified for malaria vaccine development. Of these, two leading malaria blood-stage vaccine candidates, merozoite surface protein 1 (MSP1) and apical membrane antigen 1 (AMA1), have been intensively studied as promising vaccine candidates. These two antigens are well conserved across all species of *Plasmodium* and play important roles in erythrocyte invasion and blood-stage growth. Several passive and active immunization studies have indicated that both antigens elicit protective immune responses and serve as targets for invasion-blocking antibodies [1], [2], [3].

MSP1 is synthesized as an approximately 200-kDa precursor protein at the schizont stage and is further proteolytically cleaved into a number of discrete products residing on the surface of the merozoite that invades the erythrocyte [4]. After processing, the C-terminal 19-kDa fragment (MSP1_19_) remains on the merozoite surface during erythrocyte invasion and therefore is an ideal target for blocking parasite invasion into the erythrocyte [5]. Several studies have shown that immunization with the bacterially-produced recombinant MSP1_19_ with an adjuvant completely protects mice against *P. yoelii* challenge [6], [7], [8]. The *P. falciparum* MSP1_19_ has been implicated as a target for protective immunity in a large number of studies, including seroepidemiological studies of naturally-acquired immunity, vaccination studies in non-human primates and *in vitro* cultures [9]. In particular, antibodies to MSP1_19_, either affinity purified from immune human sera or monoclonal or polyclonal experimental sera, are capable of inhibiting parasite growth *in vitro* [10], [11], [12]. Recently, however, the value of these *in vitro* assays has come into question because cytophilic MSP1_19_-specific antibodies appear to be more important for controlling infection than previously thought [13], [14] and the protective efficacy of MSP1_19_-based vaccines do not correlate with anti-MSP1_19_ antibody titers or *in vitro* parasite-inhibitory activity in animal models [15].

AMA1, synthesized as a 60–80-kDa protein during schizogony, is a microneme protein involved in merozoite invasion of erythrocytes. AMA1 possesses a large N-terminal cysteine-rich ectodomain, followed by a single transmembrane domain and a short C-terminal cytoplasmic tail. The ectodomain has been divided into three domains (I, II, and III) based on the disulfide bond position [16], [17] and the recent crystal structure [18]. Domain III binds to human erythrocytes [19] and serves as a target for growth-inhibitory antibodies [20]. Immunization with parasite-derived AMA1 and recombinant AMA1 induced significant levels of protection against *P. yoelii* challenge in mice [21] and against *P. falciparum* challenge in *Aotus* monkeys [22], respectively. Despite its promising potential, neither PfMSP1_19_- nor PfAMA1-based vaccine candidates have yet shown satisfactory outcomes in human clinical trials. Discrepancies in the vaccine efficacies observed between the *P. yoelii* model and human clinical trials still remain problematic, although poor immunogenicity and genetic polymorphisms are thought to be major obstacles for vaccine development using these molecules [23], [24], [25], [26]. 

We have recently developed a baculoviral-based vaccine (BBV) expressing PyMSP1_19_ on the surface of the viral envelope [27]. Adjuvant-free intranasal immunization with this vaccine induced not only strong systemic humoral immune responses with high titers of PyMSP1_19_-specific antibody but also natural boosting of PyMSP1_19_-specific antibody responses shortly after challenge, and conferred complete protection. As a next step, we have generated a PfMSP1_19_-BBV vaccine to address the possibility of its use in a clinical setting. In the present study, we evaluated the protective efficacies of a series of MSP1_19_- and AMA1-BBVs against challenge with transgenic *P. berghei* expressing PfMSP1_19_ as well as *P. berghei* in mice. Our results show that although immunization with these BBVs induced high levels of antigen-specific antibody titers, none of the immunized mice were protected against challenge. In contrast, immunization with PyMSP1_19_- and PyAMA1-BBVs provided 100% and 40% protection against lethal challenge with *P. yoelii*, respectively. These data suggest that *P. berghei* possesses additional molecules and/or mechanisms that circumvent the host's immune responses to MSP1_19_ and AMA1. The present study provides important insights for malaria blood-stage vaccine development using *P. yoelii* and *P. berghei*.

## Materials and Methods

### Ethics Statement

All care and handling of the animals was in accordance with the Guidelines for Animal Care and Use prepared by Jichi Medical University, following approval (ID: 09193) by the Jichi Medical University Ethical Review Board.

### Mice and parasites

Female BALB/c and C57/BL6 mice, 7 to 8 weeks of age at the start of the experiments, were purchased from Nippon Clea (Tokyo, Japan). *P. berghei* ANKA were used for challenge infection. *P. yoelii* 17XL, a lethal murine malaria parasite, was kindly provided by T. Tsuboi (Ehime University, Matsuyama, Japan). *P. falciparum* 3D7 was kindly provided by K. Kita (The University of Tokyo, Tokyo, Japan). Pb-PfM19 [28], transgenic *P. berghei* ANKA expressing PfMSP1_19_ in place of native PbMSP1_19_, was kindly provided by T. Koning-Ward (The Walter and Eliza Hall Institute of Medical research, Parkville, Australia).

### Recombinant baculovirus

For the construction of MSP1_19_-expressing baculovirus transfer vectors, the DNA sequence corresponding to amino acids Asn_1607_–Asn_1702_ of PfMSP1_19_ was amplified from *P. falciparum* 3D7 genomic DNA using the primer pair pPfMSP1_19_-F1 (5′-GAATTCAACATTTCACAACACCAATGCGTAAAAAAAC-3′)/pPfMSP1_19_-R1 (5′-CCCGGGCGTTAGAGGAACTGCAGAAAATACCATCG-3′). Similarly, the DNA sequence corresponding to amino acids Gly_1672_-Ser_1767_ of PbMSP1_19_ was amplified from *P. berghei* ANKA genomic DNA using the primer pair pPbMSP1_19_-F1 (5′-GAATTCGGTATAGACCCTAAGCATGTATGT-3′)/pPbMSP1_19_-R1 (5′-CCCGGGAGCTACAGAATACACCATCATAATATGC-3′). Each of the resulting PCR products was ligated into the *EcoR*I/*Sma*I sites of pBACsurf-PyMSP1_19_ [27] to construct baculovirus transfer vectors.

For the construction of AMA1-expressing baculovirus transfer vectors, the DNA sequences corresponding to amino acids Asn_53_-Glu_478_ (domains I, II and III) and Glu_380_-Glu_478_ (domain III) of PbAMA1 (PbAMA1-D123 and PbAMA1-D3, respectively) were amplified from *P. berghei* ANKA genomic DNA using the primer pairs pPbAMA1-F1 (5′-GAATTCAATCCATGGGAAAAGTATACGGAAAAATAT-3′)/pPbAMA1-R1 (5′-CCCGGGCTTCTCTGGTTTGATGGGCTTTCATATGCAC-3′) and pPbAMA1-F2 (5′-GAATTCGAAGAGTTCGAAGAACAATTTCCTTGTGAT-3′)/pPbAMA1-R1, respectively. Similarly, the DNA sequences corresponding to amino acids Ile_52_-Lys_479_ (domains I, II and III) and Glu_380_-Lys_479_ (domain III) of PyAMA1 (PyAMA1-D123 and PyAMA1-D3, respectively) were amplified from *P. yoelii* 17XL genomic DNA using the primer pairs pPyAMA1-F1 (5′-GAATTCAATCCATGGGATAAATATATGGAAAAATATGAT-3′)/pPyAMA1-R1 (5′-CCCGGGTTTTCTGGTTTGGGTTTTCATAGTCACCTAT-3′) and pPyAMA1-F2 (5′-GAATTCGAAGAAAATTTTCCTTGTGAAATATAT-3′)/pPyAMA1-R1, respectively. Each of the resulting PCR products was ligated into the *EcoR*I/*Sma*I sites of pBACsurf-PyMSP1_19_ [27] to construct baculovirus transfer vectors. Recombinant baculoviruses, AcNPV-PfMSP1_19_surf, AcNPV-PbMSP1_19_surf, AcNPV-PyAMA1-D123surf, AcNPV-PyAMA1-D3surf, AcNPV-PbAMA1-D123surf, and AcNPV-PbAMA1-D3surf were generated in *Spodoptera frugiperda* (Sf9) cells by co-transfection of the corresponding baculovirus transfer vector with BacVector-2000 DNA (Novagen) according to the manufacturer's protocol. AcNPV-PyMSP1_19_surf has been described previously [27]. Purification of baculovirus virions was performed as described previously [29]. The purified baculovirus particles were free of endotoxin (<0.01 endotoxin units/10^9^ pfu), as determined by an Endospecy® endotoxin measurement kit (Seikagaku Co., Tokyo, Japan).

### Recombinant proteins

The *Pfmsp1_19_*, *Pbmsp1_19_*, *Pyama1-D3* and *Pbama1-D3* genes were excised from pBACsurf-PfMSP1_19_, pBACsurf-PbMSP1_19_, pBACsurf-PyAMA1-D3, pBACsurf-PbAMA1-D3, respectively, by digestion with *EcoR*I and *Sma*I. Each of these DNA fragments was cloned into the *EcoR* I/*Sma*I sites of pGEX-4T-1 (GE Healthcare UK Limited, Buckinghamshire, UK). Recombinant PfMSP1_19_, PbMSP1_19_, PyAMA1-D3 and PbAMA1-D3 created as GST fusion proteins (termed GST-PfMSP1_19_, GST-PbMSP1_19_, GST-PyAMA1-D3 and GST-PbAMA1-D3 respectively), were expressed in *Escherichia coli* and purified using GST affinity columns (GE Healthcare UK Limited) as described previously [30]. GST-PyMSP1_19_ was used as an immunogen for vaccination and antigen for ELISA as described previously [27]. These recombinant proteins were recognized by *P. yoelii* or *P. berghei*-hyperimmune sera, which were obtained from BALB/c mice that had recovered from repeated infections of the corresponding parasite following treatment with chloroquine as described previously [28]. A recombinant MSP1_19_ (yPfMSP1_19_) of *P. falciparum*, produced in *Saccharomyces cerevisiae*, was obtained from MR4 (Manassas, VA). We confirmed that the bacterially-produced GST-PfMSP1_19_ and yPfMSP1_19_ proteins had the similar immunogenicity available in ELISA's using sera obtained from AcNPV-PfMSP1_19_surf-immunized mice and malaria-exposed individuals living in a hyperendemic area. These GST-fusion proteins were used as immunogens for vaccination and antigens for ELISA. A recombinant MSP1_19_ (yPyMSP1_19_) of *P. yoelii* 17XL, produced in *S. cerevisiae*, was obtained from MR4, and used as an antigen for ELISA.

### Immunoblotting and indirect immunofluorescence assay (IFA)

For immunoblotting, protein samples were separated on a 6% SDS-PAGE gel, transferred to Immobilon™ Transfer Membrane (Millipore, Bedford, MA). The membrane was treated either with the anti-PfMSP1_19_ mAb 5.2 (MR4, Manassas, VA), anti-gp64 mAb (BD Biosciences, Bedford, MA), or *P. berghei*- or *P. yoelii*- hyperimmune sera. Polypeptides recognized by the antibodies were visualized by color development with 5-bromo-4-chloro-3-indolylphosphate *p*-toluidine salt/nitroblue tetrazolium chloride substrate (Invitrogen) following biotinylated anti-mouse IgG secondary antibody (Vector Laboratories, Burlingame, CA) as described previously [29]. Alternatively, polypeptides recognized by the antibodies were detected with ECL™ Western Blotting Detection Reagents (GE Healthcare UK Ltd.) using an HRP-conjugated goat anti-mouse IgG (H+L) secondary antibody (Bio-Rad, Hercules, CA).

For IFA, erythrocytes infected with parasites were washed, aliquoted onto multiwell slides, and fixed in 4% paraformaldehyde or methanol/acetone (4∶6) for 30 min. Sera were diluted 1∶1,000 and incubated on the slide at room temperature for 1 h following permeabilization with 1% Triton X in PBS. After washing, the slides were incubated with fluorescein isothiocyanate (FITC)-conjugated goat anti-mouse IgG for 1 h, washed, and covered with a drop of VECTASHIELD™ with DAPI (4′ 6-diamidion-2-phenylindole) (Vector Laboratories). Bound antibodies were detected using a BZ 9000 fluorescence microscope (Keyence, Tokyo, Japan).

### Immunization and challenge infections

Mice were immunized three times at 3-week intervals with 5×10^7^ pfu of BBV either by an intramuscular (i.m.) or intranasal (i.n.) route as described previously [27]. As a comparative control, mice were immunized intraperitoneally (i.p.) with 50 µg of GST-PbMSP1_19_, GST-PfMSP1_19_ or GST-PyMSP1_19_ in 2 mg of aluminum hydroxide (Imject® Alum, Pierce) three times at 3-week intervals. For each route of immunization, 2 weeks after the final immunization, sera were collected and mice were challenged with 1,000 live parasite-infected red blood cells (pRBC) by intravenous injection. The course of parasitemia was monitored by microscopic examination of Giemsa-stained thin smears of tail blood.

### Enzyme-linked immunosorbent assay (ELISA) for antibody titers

Sera obtained from immunized mice were collected by tail bleeds 2 weeks after the final immunization prior to challenge. For some mice, serum was also collected periodically after challenge. For MSP1_19_-specific antibody detection, pre-coated ELISA plates with 100 ng/well GST-PfMSP1_19_, GST-PbMSP1_19_, GST-PyMSP1_19_, PyAMA1-D3, PbAMA1-D3, and yPyMSP1_19_ were incubated with serial dilutions of sera obtained from immunized and control mice. MSP1_19_- or AMA1D3-specific antibodies were detected using HRP-conjugated goat anti-mouse IgG (H+L) (Bio-Rad). The plates were developed with peroxidase substrate solution [H_2_O_2_ and 2,2′-azino-bis(3-ethylbenzothiazoline-6-sulfonate)]. The optical density (OD) at 414 nm of each well was measured using a plate reader. Endpoint titers were expressed as the reciprocal of the highest sample dilution for which the OD was equal or greater than the mean OD of non-immune control sera.

### Infection and drug treatment

Groups of five mice were infected with *P. yoelii* XL or *P. berghei* ANKA pRBC. When the parasitemia had reached 1–3%, mice were treated i.m. on 3 consecutive days with 100 µl of 10 mg/ml Artemether Injection® (Kunming Pharmaceutical Corp., Kunming, China) dissolved in olive oil (Yoshida Pharmaceutical Corp., Tokyo, Japan). Four weeks after the completion of the infection and drug cure regimen, mice were re-infected three times with 1,000 live pRBC of homologous parasite at 4-week intervals. Self-cured mice were challenged with 1,000 live pRBC of heterologous parasites (*P. yoelii* XL or *P. berghei* ANKA). The same experiment was repeated. The course of parasitemia was monitored by microscopic examination of Giemsa-stained thin smears of tail blood.

## Results

### Construction of MSP1_19_-BBV

**Figure 1:**
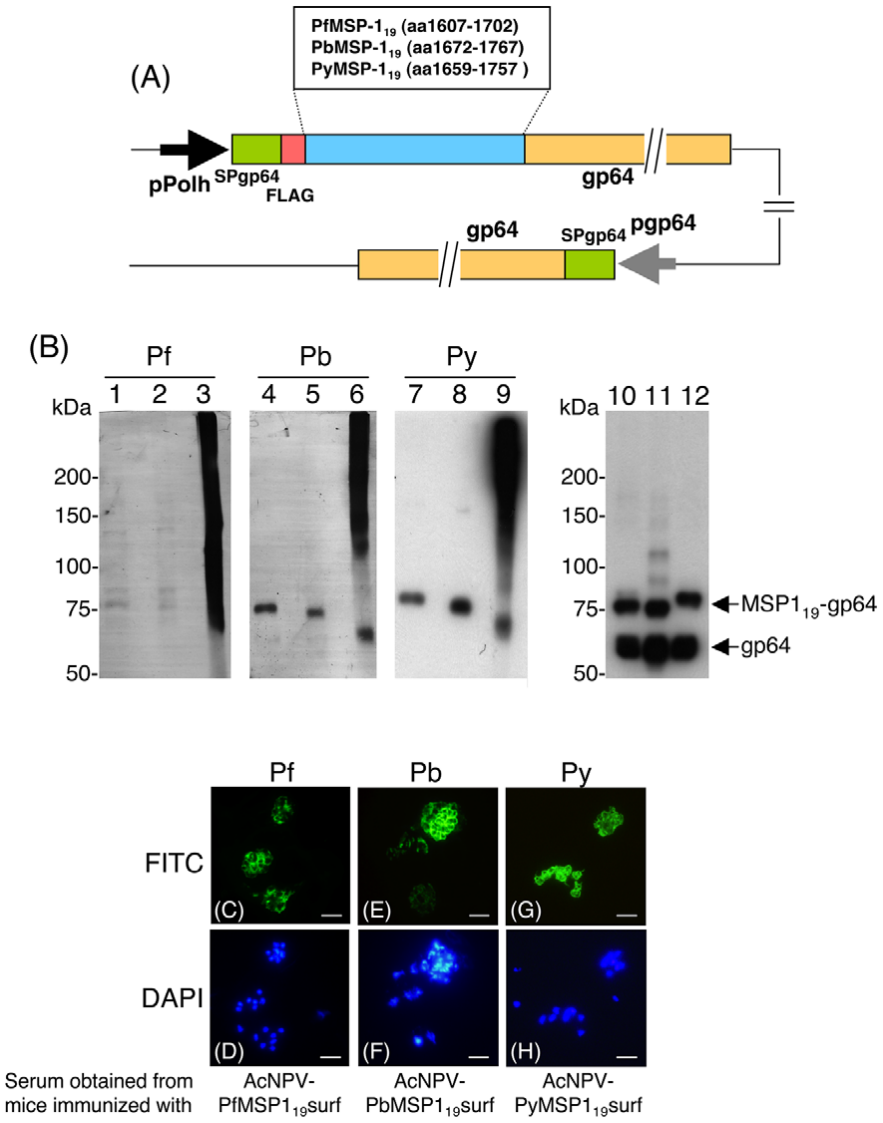
Construction and expression analysis of MSP1_19_-BBVs. (A) Schematic diagram of three MSP1_19_-BBV genomes. MSP1_19_ was expressed as a MSP1_19_-gp64 fusion protein under the control of the polyhedron promoter. Numbers indicate the amino acid positions of MSP1_19_-gp64 fusion protein and endogenous gp64 protein. pPolh, polyhedrin promoter; SP, the gp64 signal sequence; FLAG, the FLAG epitope tag; pgp64, gp64 promoter. (B) Western blot analysis of MSP1_19_-BBVs. AcNPV-PfMSP1_19_surf (lanes 1, 2, 3 and 10), AcNPV-PbMSP1_19_surf (lanes 4, 5, 6 and 11) and AcNPV-PyMSP1_19_surf (lanes 7, 8, 9 and 12) were treated with the loading buffer with 5% 2-ME (lanes 1, 4, 7, 10, 11 and 12), 0.5% 2-ME (lanes 2, 5 and 8) or without 2-ME (lanes 3, 6 and 9) and examined using the 5.2 mAb (lanes 1–3), *P. berghei*-hyperimmune serum (lanes 4–6), *P. yoelii*-hyperimmune serum (lanes 7–9) and anti-gp64 mAb (lanes 10–12). Positions of MSP1_19_-gp64 fusion protein and endogenous gp64 are shown at the right panel of lanes 10–12. (C–H) Immunofluorescence patterns of sera obtained from mice immunized with three MSP1_19_-BBVs on paraformaldehyde fixed erythrocyte smears infected with *P. falciparum* (C–D), *P. berghei* (E–F) and *P. yoelii* (G–H). The smears were incubated with serum obtained from an individual mouse immunized either with AcNPV-PfMSP1_19_surf (C), AcNPV-PbMSP1_19_surf (E) or AcNPV-PyMSP1_19_surf (G), and antibody binding was detected with secondary FITC-labeled antibody. Cell nuclei were visualized by DAPI staining on the corresponding smears (D, F and H). Scale bar, 10 µm.

Recently, we have developed a new PyMSP1_19_-BBV (AcNPV-PyMSP1_19_surf) that displays PyMSP1_19_ on the surface of the baculoviral envelope. Adjuvant-free intranasal immunization with this vaccine induced strong systemic humoral immune responses with high titers of PyMSP1_19_-specific antibody, naturally boosted the PyMSP1_19_-specific antibody response a short time after infection, and allowed 100% of mice to self-cure with very low parasitemia [27]. To apply this baculoviral vaccine system to *P. falciparum* MSP1_19_ vaccine development, we generated AcNPV-PfMSP1_19_surf and AcNPV-PbMSP1_19_surf (Figure 1A). Each construct harbored a gene cassette that consisted of the gp64 signal sequence and the MSP1_19_ gene fused to the N-terminus of the AcNPV major envelope protein gp64. Expression of these gene cassettes was driven by the polyhedrin promoter. Thus these BBVs were designed to express MSP1_19_ on the viral envelope as a gp64 fusion protein.

### MSP1_19_ fused to gp64 exhibits the three-dimensional structure of the native MSP1_19_ with correctly formed disulfide bonds

Western blotting analysis shows that the anti-PfMSP1_19_ mAb 5.2, which has previously been shown to recognize a conformation-dependent epitope [31], reacted with very faint doublet bands with relative molecular masses (M_r_) of 75 and 85 kDa in the presence of 2-ME (Figure 1B, lanes 1–2). Much stronger smear bands with high M_r_ were seen in the absence of 2-ME, (lane 3), indicating formation of oligomer complexes. *P. berghei*-hyperimmune serum reacted with a 75-kDa band corresponding to the PbMSP1_19_-gp64 fusion protein in the presence of 5% 2-ME. Similar to the PfMSP1_19_-gp64 fusion protein complex, strong smear bands of PbMSP1_19_-gp64 fusion protein with high M_r_ were seen in the absence of 2-ME (lane 6). These results are consistent with previous results showing that the PyMSP1_19_-gp64 fusion protein was susceptible to treatment with 2-ME as detected using *P. yoelii*-hyperimmune serum (lanes 7–9) [27]. The anti-gp64 mAb reacted with three MSP1_19_-gp64 fusion proteins and endogenous gp64 (lanes 10–12). The total intensity of endogenous gp64 plus each MSP1_19_-gp64 fusion protein band seems to be similar to that of each smear band (lanes 3, 6 and 9) under the non-reducing conditions. These results indicate that these three MSP1_19_-gp64 fusion proteins form oligomer complexes not only with MSP1_19_-gp64 fusion protein but also endogenous gp64 on the virus envelope and retain the three-dimensional structures of the native MSP1_19_ with correctly formed disulfide bonds.

### High level PfMSP1_19_-specifc antibody titers induced by AcNPV-PfMSP1_19_surf did not confer protection against Pb-PfMSP1 parasites

Both i.m. and i.n. immunization of BALB/c mice with AcNPV-PfMSP1_19_surf induced high titers of PfMSP1_19_-specifc antibodies (72,600±19,300 and 167,000±47,300, respectively) (Table 1, EXP1). These immune sera strongly reacted with native PfMSP1_19_ on *P. falciparum* schizonts with circumferential staining, characteristic of an antigen present on the parasite surface (Figure 1C). However, none of the immunized mice survived following challenge with Pb-PfM19 parasites (transgenic *P. berghei* expressing PfMSP1_19_ in place of native PbMSP1_19_). In accordance with our previous study [27], the i.m. and i.n. immunization with AcNPV-PyMSP1_19_surf conferred 50% and 100% protection, respectively, against *P. yoelii* challenge infection (Table 1, EXP2,G4-5). Moreover, we and others have demonstrated that mice immunized with *E. coli-*producing GST-PyMSP1_19_ formulated in Freund's or alum adjuvant were protected against *P. yoelii* challenge infection. While immunization with GST-PyMSP1_19_ plus alum provided 70% protection against *P. yoelii* challenge infection, mice similarly immunized with the same preparation of GST-PfMSP1_19_ did not survive following Pb-PfMSP1 parasite challenge, although the immunization induced high titers of PfMSP1_19_-specifc antibodies (134,000±28,600). Interestingly, one of 10 naïve mice self-cured from high parasitemia following *P. yoelii* 17XL infection (Table 1, EXP2 G1). We observed several monocytes actively phagocytosing the parasites in the blood of the self-cured mouse (Supplementary Figure S1). This is a very rare case because *P. yoelii* 17XL infection of BALB/c mice was 100% lethal in our previous experiments. The parasite clearance by phagocytosis may be due to the activation of innate immunity during infection. It would be interesting to address the triggers behind the induction of protective immunity in a naïve mouse during infection.

**Table 1:** Protective efficacies of MSP1_19_-BBVs against challenge infection*^a^*.[Table-fn nt101]

Vaccine (Challenge parasite)	Mouse strain	Route	Anti-MSP1_19_ titer^b^ mean±SE	No. of protected mice/total no. (%)
EXP1 (Pb-PfM19)				
G1: Non-immunized	BALB/c	-	ND^c^	0/5 (0)
G2: GST-PfMSP1+alum	BALB/c	i.p.	134,000±28,600	0/5 (0)
G3: AcNPV-WT	BALB/c	i.m.	ND	0/5 (0)
G4: AcNPV-PfMSP1_19_surf	BALB/c	i.m.	72,600±19,300	0/5 (0)
G5: AcNPV-PfMSP1_19_surf	BALB/c	i.n.	167,000±47,300	0/5 (0)
				
EXP2 (*P. yoelii*)				
G1: Non-immunize	BALB/c	-	ND	1/10 (10)
G2: GST-PyMSP1_19_+alum	BALB/c	i.p.	171,000±138,000	7/10 (70)
G3: AcNPV-WT	BALB/c	i.m.	ND	0/10 (0)
G4: AcNPV-PyMSP1_19_surf	BALB/c	i.m.	159,000±55,700	5/10 (50)
G5: AcNPV-PyMSP1_19_surf	BALB/c	i.n.	126,000±33,900	10/10 (100)
				
EXP3 (*P. berghei*)				
G1: Non-immunize	BALB/c	-	ND	0/5 (0)
G2: GST-PbMSP1_19_+alum	BALB/c	i.p.	256,000±55,000	0/10 (0)
G3: AcNPV-WT	BALB/c	i.m.	ND	0/5 (0)
G4: AcNPV-PbMSP1_19_surf	BALB/c	i.m.	103,000±31,700	0/5 (0)
G5: AcNPV-PbMSP1_19_surf	BALB/c	i.n.	97,800±49,800	0/10 (0)
G6: Non-immunize	C57/BL6	-	ND	0/5 (0)
G7: AcNPV-WT	C57/BL6	i.m.	ND	0/5 (0)
G8: AcNPV-PbMSP1_19_surf	C57/BL6	i.m.	88,100±8,590	0/5 (0)

a Groups of mice were immunized with MSP1_19_-BBVs three times and challenged either with Pb-PfMSP19, *P. yoelii* or *P. berghei* following blood collection for ELISA.

b Levels of PfMSP1_19_-, PyMSP1_19_- and PbMSP1_19_- specific IgG for EXP1, 2 and 3, respectively, were measured by ELISA.

c ND, not detectable level (<500).

**Figure 2:**
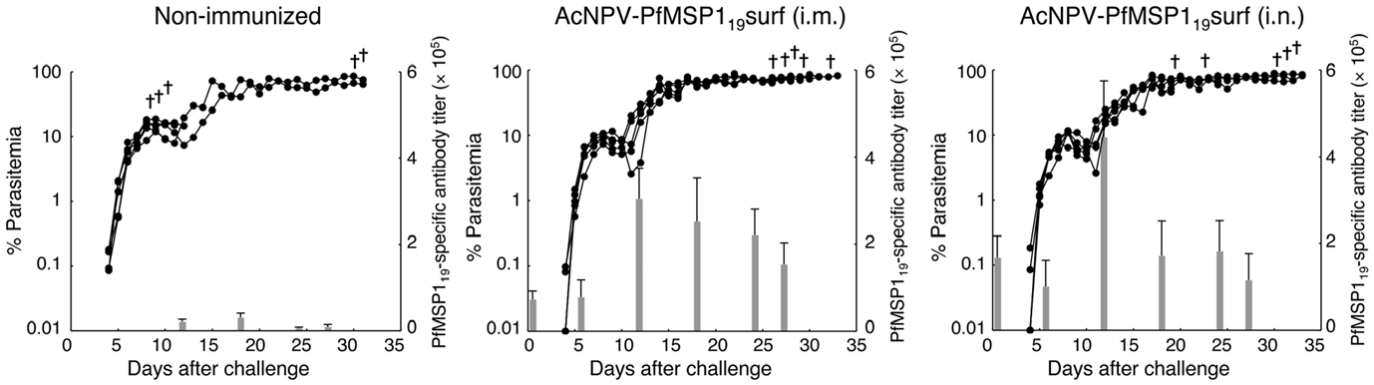
Kinetics of PfMSP1_19_-specific antibody titers and parasitemia during the course of infection. Groups of mice were non-immunized or immunized either i.m. or i.n. with AcNPV-PfMSP1_19_surf, and then challenged i.v. with 10^3^ Pb-PfM19 pRBC. Parasitemia was monitored daily 4 days after challenge and sera were collected periodically post-challenge to measure antibody titers. The bar chart indicates PfMSP1_19_-specific antibody titers on the left vertical axis. The line graph indicates the course of parasitemia (%) on the right vertical axis. (+), death.

To examine whether natural boosting of PfMSP-1_19_-specific antibodies was induced, the kinetics of the PfMSP1_19_-specific antibody titers and parasitemia during the course of infection were determined. PfMSP1_19_-specific antibodies induced by i.m. and i.n. immunization with AcNPV-PfMSP1_19_surf increased 2.7- and 4.2-fold 11 days after challenge infection (Figure 2), indicating natural boosting by challenge infection. However, the immunized groups died with high levels of parasitemia and anemia but without signs of cerebral malaria, which is similar to the non-immunized group.

### AcNPV-PbMSP1_19_surf was ineffective against *P. berghei*

To address the possibility that *P. berghei* is resistant to immune responses to PbMSP1_19_, AcNPV-PbMSP1_19_surf was generated with a construct similar to AcNPV-PyMSP1_19_surf and AcNPV-PfMSP1_19_surf. As for the Pb-PfMSP1 parasites, none of the BALB/c mice immunized i.m or i.n. with AcNPV-PbMSP1_19_surf survived following *P. berghei* challenge (Table 1, EXP3 G4–5), although immunization induced high titers of PbMSP1_19_-specifc antibodies (103,000±31,700 and 97,800±49,800, respectively) with strong reactivity against *P. berghei* mature schizonts (Figure 1E). In addition, none of the BALB/c mice immunized with GST-PbMSP1_19_ plus alum survived following *P. berghei* challenge, although the immunization induced high titers of PbMSP1_19_-specifc antibodies (256,000±55,000). There is no difference in the course of infection and survival time between the AcNPV-PbMSP1_19_ and non-immunized BALB/c groups (Supplementary Figure S2). Since *P. berghei* ANKA infection of C57BL/6 mice, but not BALB/c mice, has been shown to lead to “cerebral malaria” [32], it is important to examine whether the vaccine efficacy and the course of infection are different between BALB/c and C57BL/6 mice. All groups of C57BL/6 mice infected with *P. berghei* ANKA (Table 1, EXP 3 G6–8) died exhibiting low parasitemia (<15%) 8–10 days after challenge, which may be due to cerebral malaria (Supplementary Figure S2). Similar to BALB/c mice, there is no difference in the course of infection and survival time between AcNPV-PbMSP1_19_ and non-immunized B57BL/6 groups (Supplementary Figure S2). Thus AcNPV-PbMSP1_19_ did not contribute to any protective effect or reduction of symptoms either in BALB/c or C57BL/6 mice, indicating that *P. berghei* and Pb-PfM19 parasites circumvent immune responses to MSP1_19_-BBVs, which are effective for *P. yoelii*.

### Construction and expression of AMA1-BBV

**Figure 3:**
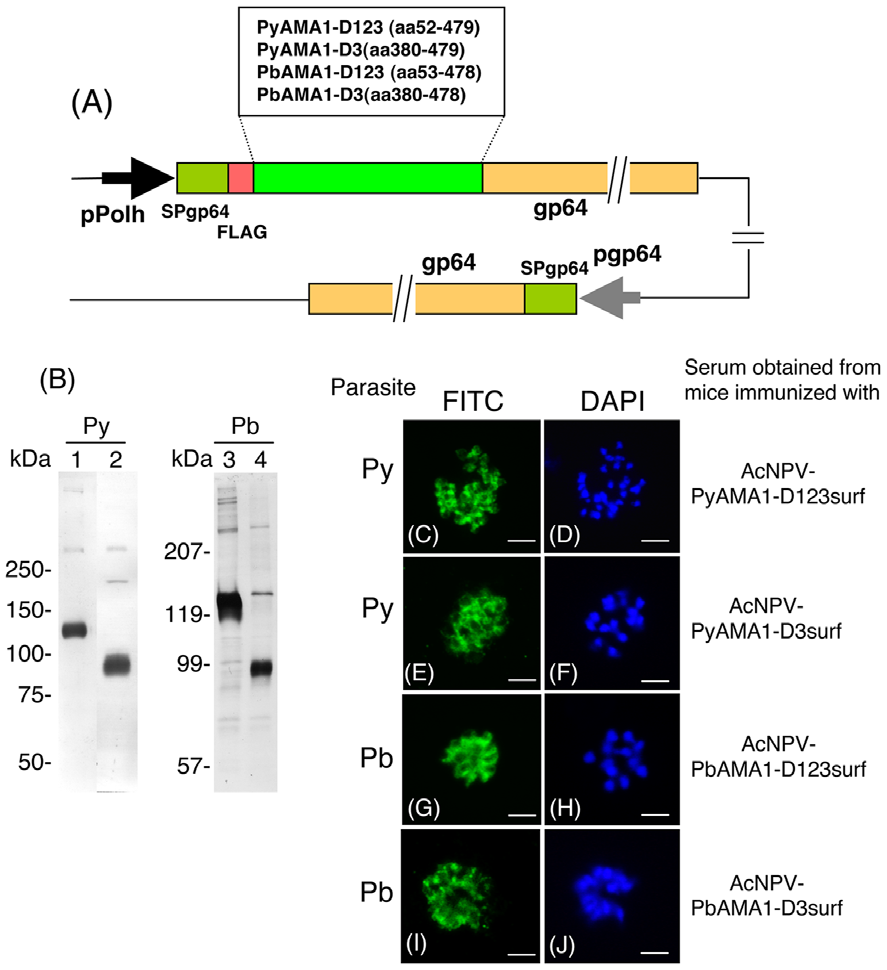
Construction and expression analysis of AMA1-BBVs. (A) Schematic diagram of four AMA1-BBV genomes. AMA1 was expressed as an AMA1-gp64 fusion protein under the control of the polyhedron promoter. Numbers indicate the amino acid positions of AMA1-gp64 fusion protein and endogenous gp64 protein. pPolh, polyhedrin promoter; SP, the gp64 signal sequence; FLAG, the FLAG epitope tag; pgp64, gp64 promoter. (B) Western blot analysis of AMA1-BBVs. AcNPV-PyAMA1-D123surf (lane 1), AcNPV-PyAMA1-D3surf (lane 2), AcNPV-PbAMA1-D123surf (lane 3) and AcNPV-PbAMA1-D3surf (lane 4) were treated with the loading buffer containing 1% 2-ME and examined using *P. yoelii*-hyperimmune serum (lanes 1 and 2), or *P. berghei*-hyperimmune serum (lanes 3 and 4). (C–J) Immunofluorescence patterns of sera obtained from mice immunized with four AMA1-BBVs on methanol-acetone fixed smears of erythrocytes infected with *P. yoelii* (C and E) and *P. berghei* (G and I). The smears were incubated with serum obtained from an individual mouse immunized either with AcNPV-PyAMA1-D123surf (C), AcNPV-PyAMA1-D3surf (E), AcNPV-PbAMA1-D123surf (G) or AcNPV-PbAMA1-D3surf (I), and antibody binding was detected with a secondary FITC-labeled antibody. Cell nuclei were visualized by DAPI staining on the corresponding smears (D, F, H and J). Scale bar, 10 µm.

To compare the protective efficacies of another leading vaccine candidate, AMA1, against *P. yoelii* and *P. berghei*, we generated two kinds of PyAMA1- and PbAMA1-BBVs consisting of ectodomains I-III or III alone (AcNPV-PyAMA1-D123surf, AcNPV-PyAMA1-D3surf, AcNPV-PbAMA1-D123suf, and AcNPV-PbAMA1-D3surf) (Figure 3A). Similar to MSP1_19_-BBV, each construct harbored a gene cassette that consisted of the gp64 signal sequence and the target gene (PyAMA1-D123, PyAMA1-D3, PbAMA1-D123, and PbAMA1-D3) fused to the N-terminus of the AcNPV major envelope protein gp64. Western blotting analysis shows that *P. yoelii*-hyperimmune serum reacted with the PyAMA1-D123- and PyAMA1-D3-gp64 fusion proteins of AcNPV-PyAMA1-D123suf and AcNPV-PyAMA1-D3surf with molecular weights of 125 kDa and 95 kDa, respectively (Figure 3B, lanes 1 and 2). Similar results were obtained with the PbAMA1-D123- and PbAMA1-D3-gp64 fusion proteins of AcNPV-PbAMA1-D123suf and AcNPV-PbAMA1-D3surf against *P. berghei*-hyperimmune serum (lanes 3 and 4).

### Protective efficacy of AMA1-BBV against challenge infection

Intramuscular immunization of BALB/c mice with AcNPV-PyAMA1-D123surf induced higher titers of PyAMA1-specifc antibodies than i.n. immunization (i.m. vs. i.n.  = 20,900±8,700 vs. 7,200±2,470) (Table 2, EXP4 G2–3). Both immune sera strongly reacted with native PyAMA1 on *P. yoelii* mature schizonts, which is consistent with AMA1 localization on the surface of merozoites (Figure 3C and E). In the i.m. AcNPV-PyAMA1-D123surf group, five of 10 mice (50%) survived *P. yoelii* challenge infection, whereas two of 10 mice (20%) survived challenge infection in the i.n. AcNPV-PyAMA1-D123surf group. Although both i.m. and i.n. immunization with AcNPV-PyAMA1-D3surf induced similar levels of PyAMA1-specifc antibodies, all immunized mice died following *P. yoelii* challenge infection, indicating that PyAMA1-D123 induced partial protective immune responses, but not PyAMA1-D3. In spite of correct folding on the surface of the baculoviral virion, PyAMA1-D3 may not have any neutralizing epitopes that protect against *P. yoelii* infection. Consistent with our previous study [27], the i.m. immunization (40% protection) with AcNPV-PyMSP1_19_surf was less effective than the i.n. immunization (100% protection). Interestingly, when mice were immunized i.m. with a mixture of AcNPV-PyMSP1_19_surf and AcNPV-PyAMA1-D123surf, all mice induced PyMSP1_19_- and PyAMA1-specific antibodies and survived *P. yoelii* challenge infection with less severe infection outcomes and low peak parasitemia, indicating a synergistic effect of PyMSP1_19_ and PyAMA1 on protection. In contrast to the *P. yoelii* model, two PbAMA1-BBVs (AcNPV-PbAMA1-D123surf and AcNPV-PbAMA1-D3surf) failed to protect against *P. berghei* challenge, although PbAMA1-specific antibodies, which can recognize *P. berghei* schizonts (Figure 3G and I), were induced (Table 2, EXP5).

**Table 2:** Protective efficacies of AMA1-BBVs against challenge infection*^a^*.[Table-fn nt104]

Vaccine (Challenge parasite)	Route	Anti-AMA1 D3 antibody titer^c^ mean±SE	Anti-MSP1_19_ antibody titer^c^ mean±SE	No. of protected mice/total no. (%)
EXP4 (*P. yoelii*)				
G1: Non-immunized	-	ND^d^	ND	0/5 (0)
G2: AcNPV-PyAMA1-D123surf	i.m.	20,900±8,700	ND	5/10 (50)
G3: AcNPV-PyAMA1-D123surf	i.n.	7,200±2,470	ND	2/10 (20)
G4: AcNPV-PyAMA1-D3surf	i.m.	26,100±6,350	ND	0/5 (0)
G5: AcNPV-PyAMA1-D3surf	i.n.	12,300±2,450	ND	0/5 (0)
G6: AcNPV-PyMSP1_19_surf	i.m.	ND	70,800±11,210	2/5 (40)
G7^b^: AcNPV-PyAMA1-D123surf + AcNPV-PyMSP1_19_surf	i.m.	15,200±5,800	94,600±15,720	5/5 (100)
EXP5 (*P. berghei*)				
G1: Non-immunized	-	ND	ND	0/5 (0)
G2: AcNPV-PbAMA1-D123surf	i.m.	9,800±3,010	ND	0/5 (0)
G3: AcNPV-PbAMA1-D3surf	i.m.	16,900±5,310	ND	0/5 (0)

a Groups of BALB/c mice were immunized with AMA1-BBVs three times and challenged either with *P. yoelii* or *P. berghei* following blood collection for ELISA.

b The two BBVs (AcNPV-PyAMA1-D123surf and AcNPV-PyMSP1_19_surf) were mixed (2.5×10^7^ pfu each) and used for immunization.

c Levels of PyAMA1D3 and PyMSP1_19_- specific IgGs and PbAMA1D3 and PbMSP1_19_- specific IgGs for EXP4 and 5, respectively, were measured by ELISA.

d ND, not detectable level (<500).

### Naturally acquired protective immunity to *P. berghei* confers resistance to *P. yoelii*, but not vice versa

While many studies have consistently shown that the ELISA titer of MSP1_19_- and AMA1-immunized mice correlates with protective immunity against *P. yoelii* challenge, the corresponding *P. berghei* vaccines failed to protect against *P. berghei*. Therefore, it was of interest to determine the degree of heterologous immunity occurring between *P. yoelii* and *P. berghei*. BALB/c mice infected either with *P. yoelii* or *P. berghei* were drug-cured by three doses of Artemether. This drug treatment regimen completely cleared parasitemia so that no recurrence or recrudescence parasite appeared. The self-cured mice were re-infected three times with the homologous parasite. At the first challenge after drug treatment, some mice developed low levels of parasitemia (<10%) in both groups, and all self-cured within 7 days (data not shown). No parasitemia appeared following the second and third homologous challenges, indicating that mice naturally acquired sterile protective immunity against homologous infection. Subsequently, these mice were challenged with the heterologous parasite. All of the self-cured mice from *P. berghei* completely protected against *P. yoelii* with undetectable levels of parasitemia (Table 3, EXP6 G1), indicating the acquisition of cross-resistance to *P. yoelii* infection. In contrast, all the self-cured mice from *P. yoelii* suffered from severe courses of *P. berghei* infection with high maximum parasitemia levels (>70% parasitemia) and 100% mortality (G2). Thus naturally acquired sterile immunity induced by drug treatment and repeated *P. berghei* infection can persistently protect mice against *P. yoelii* infection, but not vice versa.

**Table 3:** Protective efficacies against heterologous challenge following drug treatment and 3 homologous re-infections*^a^*.[Table-fn nt108]

Group	Parasite used for drug treatment and re-infection	Parasite used for heterologous challenge	No. of protected mice/total no. (%)
EXP6			
G1	Pb^b^	Py	10/10 (100)
G2	Py^c^	Pb	0/10 (0)
G3	NT^d^	Pb	0/5 (0)
G4	NT	Py	0/5 (0)

a *P. berghei*- or *P. yoelii*-infected BALB/c mice were treated with Artemether®. The drug-cured mice were re-infected three times with homologous parasites at 4-week intervals. All mice survived these re-infections. The self-cured mice were then challenged with heterologous parasites.

b Pb, *P. berghei* ANKA.

c Py, *P. yoelii* 17XL.

d NT, neither drug-treatment nor infection.

## Discussion

In the present study, we demonstrate that the two leading malaria blood-stage vaccine candidate antigens, MSP1_19_ and AMA1, failed to protect against *P. berghei* and its transgenic parasite challenge infection, although immunization with these vaccines induced high levels of antigen-specific antibody titers, and the immune sera strongly reacted with the blood-stage parasites.

Our previous study showed that immunization with AcNPV-PyMSP1_19_surf completely clears *P. yoelii* shortly after challenge by a quick natural boosting response [27]. On the other hand, immunization with the bacterially-produced GST-PyMSP1_19_ plus alum impairs *P. yoelii* growth at the time of infection by induced PyMSP1_19_-specific antibodies, resulting in a delay in the onset of a patent parasitemia and protracted period of parasite inhibition [6], [33], [34], [35]. Therefore, these two vaccine formulas inducing different protective immune responses are suitable immunogens to investigate whether MSP1_19_-specific immune responses could confer protection or affect the course of infection in *P. berghei* ANKA and its transgenic models. Since MSP1_19_ is highly structured on the surface of merozoites, folding into two epidermal growth factor-like domains [36], development of MSP1_19_-based vaccines with its native three-dimensional structure would be necessary to induce protective immune responses. This is similar to PyMSP1_19_, PfMSP1_19_ and PbMSP1_19_ displayed on BBVs, which are stabilized by disulfide bonds, as evidenced by the loss of conformational mAb and hyperimmune IgG binding, respectively, in immunoblots with reduced MSP1_19_-BBVs (Figure 1B). Additional evidence of the correct structure includes the induction of IFA-reactive antibodies (Figures 2C–H). In the *P. berghei* transgenic parasite model, although the AcNPV-PfMSP1_19_surf group elicited natural boosting of vaccine-induced PfMSP1_19_-specific antibody responses during infection, there was no significant reduction of parasitemia or prolonging of survival time compared to non-immunized groups. Similarly, neither PbMSP1_19_- nor PbAMA1-BBV was effective against *P. berghei*. In addition, the bacterially-produced GST-PbMSP1_19_ protein with alum adjuvant conferred no protection. Thus *P. berghei* and its transgenic parasites completely circumvent immune responses induced by MSP1_19_- and AMA1-based vaccines.

One possible mechanism by which the growth of *P. berghei* cannot be controlled by high levels of PbMSP1_19_- or PbAMA1-specific antibodies is that *P. berghei* possesses additional molecules and/or mechanisms other than MSP1_19_ and AMA1 for erythrocyte invasion that are lacking in *P. yoelii*. In support of this, we found that when mice self-cured from *P. berghei* infection by drug treatment and subsequent *P. berghei* re-infections, they naturally acquired sterile immunity against *P. yoelii* as well as *P. berghei*, but not vice versa. This is consistent with a previous report that protective cross-immunity operates only in one direction (*P. berghei*→*P. yoelii*) since mice immunized with a formalin-fixed blood-stage *P. yoelii* were fully susceptible to *P. berghei* [37]. *P. falciparum* has been shown to have a number of invasion pathways and to be capable of entering erythrocytes by sialic acid-dependent and -independent pathways [38]. While no such parallel invasion pathways have been described for rodent malaria parasites, it is possible that the multiplicity of merozoite surface proteins in *P. berghei* may reflect involvement in alternative pathways of invasion. Although a transgenic *P. berghei* line expressing PyMSP1_19_ in place of native PbMSP1_19_ would be a good model to address this possibility, we have failed to generate the transgenic line using similar methodology for the construction of Pb-PfM19 [28], suggesting that PbMSP1_19_ and PfMSP1_19_ share similar functions essential for growth and/or invasion, but not with PyMSP1_19_.

Another possible mechanism is that *P. berghei* infection may impair antibody-dependent cellular inhibition of parasite growth. We have previously demonstrated that a genetically engineered bispecific single-chain antibody targeted to human CD3 and PfMSP1_19_ promotes merozoite phagocytosis and growth inhibition in *in vitro P. falciparum* culture through cooperation with T cells and monocytes [39]. Evidence has been accumulated to suggest that antibody action via Fc interaction with monocytes/macrophages plays an important role in protective immunity against blood-stage parasites [14], [40], [41]. In the case of the *P. yoelii* model, MSP1_19_- and AMA1-specific antibodies may effectively function in the parasite killing by cooperation with monocytes/macrophages. However, *P. berghei* infection may strongly suppress monocyte/macrophage activation through Fc receptors with MSP1_19_- or AMA1-specific antibodies. If *P. falciparum* uses mechanisms similar to those utilized by *P. berghei* to circumvent MSP1_19_- and AMA1-based vaccine properties, *P. berghei* rather than *P. yoelii* would be a more useful model to identify and evaluate new blood-stage vaccine candidate antigens for *P. falciparum*.

Recently, *P. berghei* has been genetically engineered to express representative vaccine candidate antigens (e.g., PfMSP1_19_, PfAMA1, PfCSP, Pfs25 and Pvs25) from the human malaria parasites, *P. falciparum* and *P. vivax* [28], [42], [43], [44], [45]. These transgenic *P. berghei* parasites provide great potential to investigate the protective efficacies of vaccine candidates against the human malaria parasites *in vivo*. Very recently, we have shown that transmission blocking vaccines using the BBV system can induce a remarkable reduction of malaria transmission to mosquitoes, directly evaluated by immunization of mice following challenge with Pfs25- or Pvs25-expressing *P. berghei* [46], [47]. Although *P. berghei* transgenic parasites expressing PfMSP1_19_ and PfAMA1 have been used to evaluate the inhibitory effects on parasite growth *in vitro* and through passive immunization [15], [28], [48], *in vivo* challenge experiments following active immunization have not been reported. Unlike *P. yoelii*, *P. berghei* has not been used for the evaluation of vaccine efficacy against blood-stage parasites, although the parasite has been well-studied not only for pre-erythrocytic vaccine development but also a cerebral malaria model [49]. To date, the usefulness of the transgenic *P. berghei* model for evaluating protective efficacies of blood-stage antigens remains unclear. In the present study, we were unable to show the usefulness of PfMSP1_19_-BBV as a promising malaria vaccine candidate using the transgenic *P. berghei*, although a similar construct of PyMSP1_19_-BBV provided 100% protection in the *P. yoelii* model [27]. We still cannot exclude the possibility that the absence of protection induced by the PfMSP1_19_ vaccine formulations is due to qualitative differences between the PyMSP1_19_ and PfMSP1_19_ vaccine formulations used here, rather than differences in parasite susceptibility to anti-MSP1_19_ responses, although western blot and immunologiocal analyses show that the three MSP1_19_-gp64 fusion proteins as well as GST- MSP1_19_ proteins were expressed at quantitatively similar levels and immunization with these proteins induced high titers of MSP1_19_-specific antibodies with strong reactivity against mature schizonts of the corresponding parasites. Further experiments using PfMSP1-based vaccines with GMP level, which have been evaluated in human clinical trials, would be needed to investigate the susceptibility of Pb-PfMSP1_19_ to anti-PfMSP1_19_ responses.

Our results provide important insights for malaria blood-stage vaccine development using *P. yoelii* and *P. berghei* and highlight the need to deeply investigate the relationship between rodent and human parasites. Obviously, it is important to elucidate the discrepancy in the vaccine efficacies observed between the *P. yoelii* model and human clinical trials. A better understanding of the precise relationship between rodent and human parasites should lead to a transfer of information from transgenic *P. berghei* to human vaccine development prior to human clinical settings.

## Supporting Information

Figure S1 Photomicrographs of Giemsa-stained thin blood smears of the self-cured mouse. Ten non-immunized mice were infected with *P. yoelii* 17XL-pRBC by i.v. injection. The course of parasitemia was monitored daily from 4 days post-challenge by microscopic examination of Giemsa-stained thin blood smears obtained from tail bleeds. One of these mice self-cured from high parasitemia of *P. yoelii* infection. The mouse cleared the parasites 21 days after challenge. The photomicrographs of the self-cured mouse were taken at 19, 21 and 24 days after challenge. Arrows indicate malaria pigment in monocytes phagocytosing the parasites. Original magnification, ×1,000.
(6.18 MB TIF)

Figure S2 The course of parasitemia. BALB/c and C57BL/6 mice were immunized i.m. with AcNPV-PbMSP1_19_surf or AcNPV-WT and challenged i.v. with 10^3^ *P. berghei*-pRBC. Parasitemia was monitored daily from 5 days post-challenge. All groups of BALB/c and C57BL/6 mice died 18 and 10 days after challenge, respectively. Data (mean Â±SD) are from the BALB/c (EXP3 G 1, 3 and 4) and C57BL/6 (EXP3 G6–8) shown in Table 1. closed triangle, non-immunized; open square, AcNPV-WT; closed circle, AcNPV-PbMSP1_19_surf.
(6.11 MB TIF)
