# Supplementary figures and images for: Plasmodium berghei Circumvents Immune Responses Induced by Merozoite Surface Protein 1- and Apical Membrane Antigen 1-Based Vaccines
Source: PLoS One. 2010 Oct 28;5(10):e13727. doi: 10.1371/journal.pone.0013727 (PMC2965677; doi:10.1371/journal.pone.0013727)

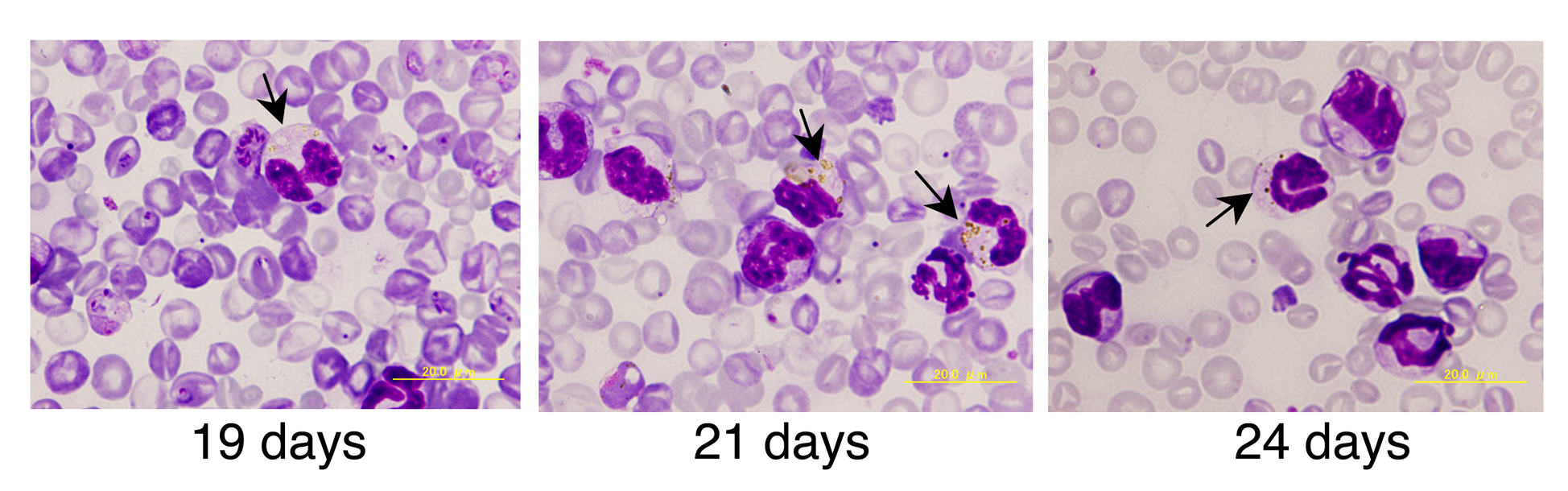

Supplement: Figure S1 — Photomicrographs of Giemsa-stained thin blood smears of the self-cured mouse. Ten non-immunized mice were infected with P. yoelii 17XL-pRBC by i.v. injection. The course of parasitemia was monitored daily from 4 days post-challenge by microscopic examination of Giemsa-stained thin blood smears obtained from tail bleeds. One of these mice self-cured from high parasitemia of P. yoelii infection. The mouse cleared the parasites 21 days after challenge. The photomicrographs of the self-cured mouse were taken at 19, 21 and 24 days after challenge. Arrows indicate malaria pigment in monocytes phagocytosing the parasites. Original magnification, ×1,000. (6.18 MB TIF) [file pone.0013727.s001.tif]

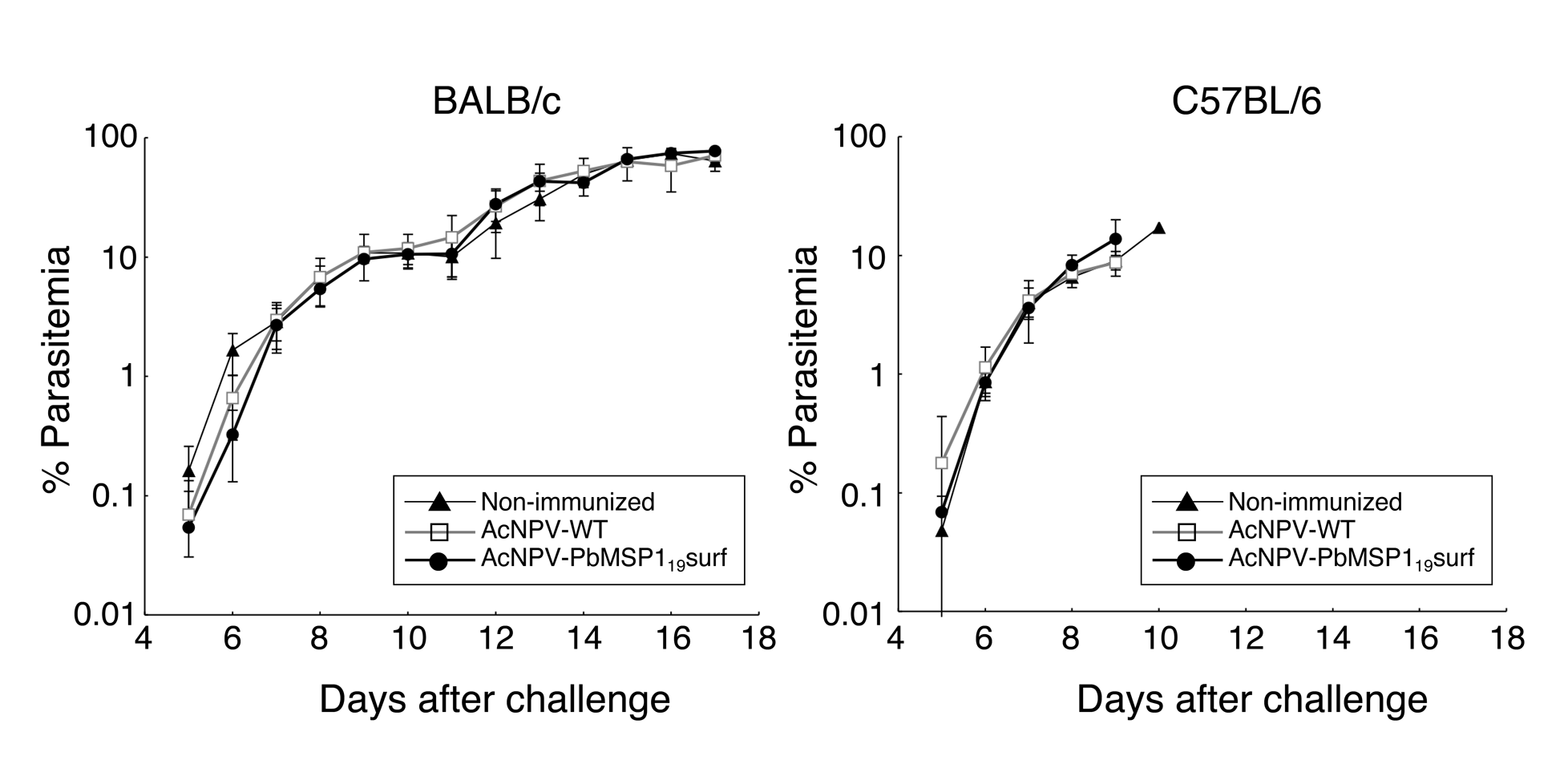

Supplement: Figure S2 — The course of parasitemia. BALB/c and C57BL/6 mice were immunized i.m. with AcNPV-PbMSP119surf or AcNPV-WT and challenged i.v. with 103 P. berghei-pRBC. Parasitemia was monitored daily from 5 days post-challenge. All groups of BALB/c and C57BL/6 mice died 18 and 10 days after challenge, respectively. Data (mean Â±SD) are from the BALB/c (EXP3 G 1, 3 and 4) and C57BL/6 (EXP3 G6–8) shown in Table 1. closed triangle, non-immunized; open square, AcNPV-WT; closed circle, AcNPV-PbMSP119surf. (6.11 MB TIF) [file pone.0013727.s002.tif]
